# Value of the surgeon’s sightline on hologram registration and targeting in mixed reality

**DOI:** 10.1007/s11548-020-02263-3

**Published:** 2020-09-28

**Authors:** Javier A. Luzon, Bojan V. Stimec, Arne O. Bakka, Bjørn Edwin, Dejan Ignjatovic

**Affiliations:** 1 Institute of Clinical Medicine, Faculty of Medicine, University of Oslo, Oslo, Norway; 2 Division of Surgery, Department of Digestive Surgery, Akershus University Hospital, Att.: Campus Ahus, 1478 Lørenskog, Norway; 3 Faculty of Medicine, Teaching Unit, Anatomy Sector, University of Geneva, Geneva, Switzerland; 4 The Intervention Centre, Oslo University Hospital, Oslo, Norway; 5 Department of Hepatopancreatobiliary Surgery, Oslo University Hospital-Rikshospitalet, Oslo, Norway

**Keywords:** Image-guided surgery, Augmented reality, Computer-assisted surgery, 3D modeling, Three-dimensional imaging, Colorectal surgery

## Abstract

**Purpose:**

Mixed reality (MR) is being evaluated as a visual tool for surgical navigation. Current literature presents unclear results on intraoperative accuracy using the Microsoft HoloLens 1^®^. This study aims to assess the impact of the *surgeon’s sightline* in an inside-out marker-based MR navigation system for open surgery.

**Methods:**

Surgeons at Akershus University Hospital tested this system. A custom-made phantom was used, containing 18 wire target crosses within its inner walls. A CT scan was obtained in order to segment all wire targets into a single 3D-model (hologram). An in-house software application (CTrue), developed for the Microsoft HoloLens 1, uploaded 3D-models and automatically registered the 3D-model with the phantom. Based on the surgeon’s sightline while registering and targeting (free sightline /F/or a strictly perpendicular sightline /P/), 4 scenarios were developed (FF-PF-FP-PP). Target error distance (TED) was obtained in three different working axes-(*XYZ*).

**Results:**

Six surgeons (5 males, age 29–62) were enrolled. A total of 864 measurements were collected in 4 scenarios, twice. Scenario PP showed the smallest TED in *XYZ*-axes mean = 2.98 mm ± SD 1.33; 2.28 mm ± SD 1.45; 2.78 mm ± SD 1.91, respectively. Scenario FF showed the largest TED in *XYZ*-axes with mean = 10.03 mm ± SD 3.19; 6.36 mm ± SD 3.36; 16.11 mm ± SD 8.91, respectively. Multiple comparison tests, grouped in scenarios and axes, showed that the majority of scenario comparisons had significantly different TED values (*p* < 0.05). *Y*-axis always presented the smallest TED regardless of scenario tested.

**Conclusion:**

A strictly perpendicular working sightline in relation to the 3D-model achieves the best accuracy results. Shortcomings in this technology, as an intraoperative visual cue, can be overcome by sightline correction. Incidentally, this is the preferred working angle for open surgery.

## Introduction

The potential of mixed reality (MR) has recently gained more attention in multiple surgical fields together with the appearance of Microsoft HoloLens 1^®^, as this device is commercially available and its technology can add new layers of anatomical information to the working field [1–4]. There are, nevertheless, several elements to consider when applying this technology in surgery, starting with the type segmentation techniques used to create anatomically correct 3D-models [5, 6], and ending with the correct *modus operandi* of the mixed reality device in question [7].

At the same time, some accuracy studies on the HoloLens have been disappointing when performing high-precision manual tasks, thus highlighting the limitations of this device and deeming it insufficient [8, 9]. From the clinical perspective, surgeons respect the *intention*-*to*-*treat* principle which entails removal of all tumor spreading pathways at surgery [10]. In bowel cancer surgery, the fatty tissue connecting the bowel to the body (the mesentery) contains all of these pathways together with vital blood vessels, which are mostly concealed by the thickness of the fat [11]. This visual obstacle is the main reason why most of the compromises in quality of surgery are made, due to higher risks for bleedings and other complications [12]. Yet, the growing body of scientific evidence states that more extensive surgery (e.g., extended lymphadenectomy) can provide better disease-free survival for patients [13, 14]. For these reasons, it seems that a viable surgical navigation system could facilitate more extensive surgery without compromising safety [15], provided acceptable accuracy. From a technical perspective, we mainly find heterogenous results in the literature describing the accuracy of the HoloLens, using mathematical methods [16, 17]. This is why we introduce in this study a perceptual variable: *the surgeon’s sightline*.

The aim of the study is to assess the value of the surgeon’s sightline on registration and targeting accuracy while using an inside-out marker-based mixed reality navigation system for open surgery.

## Materials and methods

### Experimental setup

We aim to simulate the working environment of an open right colectomy with extended lymphadenectomy [18, 19]. This operative technique refers to the extended dissection of lymph nodes (lymphadenectomy) which are hidden and embedded inside the fat layers of the mesentery. This type operation requires close proximity to large and vital vessels in order to achieve lymph node harvesting during large bowel removal in colon cancer patients. The experimental setup was created with the following elements:

- *The mixed reality navigator for open surgery* (MRNOS) phantom,
- one Microsoft HoloLens 1®,
- the “CTrue” app,
- a 3D model (hologram),
- a study subject (surgeon),
- fiducial markers for automatic registration,
- metal needles and a height-adjustable table.

These elements were applied under normal office lighting settings (140-250 LUX).

#### MRNOS: “The mixed reality navigator for open surgery” phantom

**Fig. 1:**
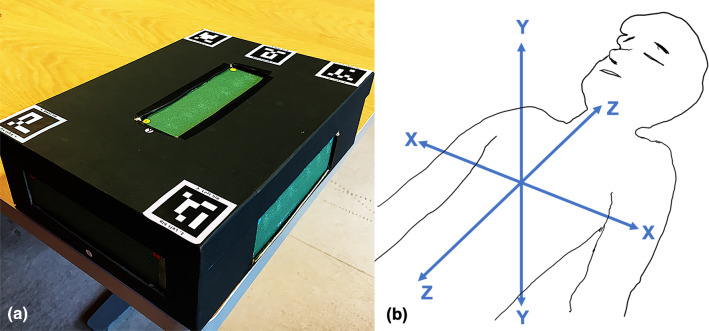
Mixed reality navigator for open surgery “MRNOS” phantom. **a** Angle picture of phantom with *XYZ*-axes visible. ArUco markers on top of phantom. **b** Represents the CT-coordinate system used in the phantom. *X*-axis: right to left, *Y*-axis: anterior to posterior and *Z*-axis: superior to inferior, also known as the RAS/LPI nomenclature

The alignment accuracy between the virtual and the physical models was tested in this custom-made phantom as shown in Fig. 1a.

**Fig. 2:**
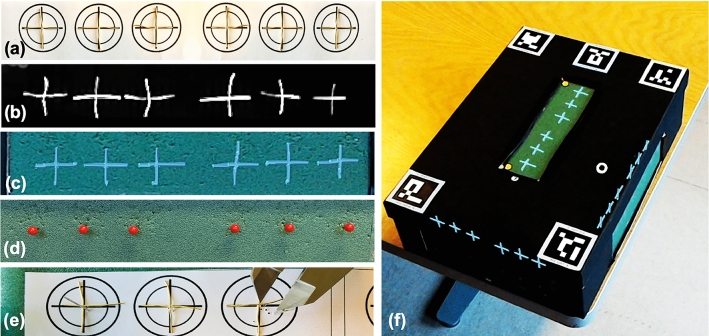
Overview of experimental workflow. **a** Anatomical internal targets. Wire crosses attached at the inner walls of the MRNOS. **b** Wire targets visible on CT scan dataset. **c** 3D model “Hologram” of corresponding wire crosses visible from the outside of the MRNOS. **d** Needle pins penetrated the 0.8 mm foam to meet the target with the guidance of the hologram. **e** View of the needle and the target from the inner wall of the MRNOS. Digital Caliper visible. **f** Hologram automatically registered/embedded into the MROS before the targeting task

A cardboard box with lid (25 × 35 × 10 cm) with three rectangular windows (17.5 × 5 cm) created on walls corresponding to the three axes (*X,Y,Z*) of the CT scan coordinate system. *X*-axis: right to left, *Y*-axis: anterior to posterior and *Z*-axis: superior to inferior, also known as the RAS/LPI nomenclature [20], as shown in Fig. 1b. Green poly-ethylene–vinyl acetate (PEVA) foam boards were modeled and used to cover these three windows. Six paper-printed target circles (20 mm in diameter), containing radiologically visible copper wire crosses aligned to these targets, were attached on the inner side of each foam board in order to hide these “anatomical targets” from the participant, as shown in Fig. 2a. Copper wires had a diameter of 0.40 mm. This gave a total of 18 wire crosses attached to 18 target circles, 6 per axis, positioned on 3 inner walls of the box. In this way, these targets acted as “anatomical internal structures” which needed to be visualized with the aid of their holographic (virtual) counterparts (Fig. 2c). These target structures were positioned at a depth of 8 mm on each axis of the MRNOS’s inner walls. Altogether, this setup allows the mimicking of the actual surgical scenario where structures of interest can be embedded under an 8 mm layer of mesenteric fat as shown in Fig. 2f.

#### Hardware: Microsoft HoloLens

The commercially available inside-out optical see-through (OST) head-mounted display (HMD) called Microsoft HoloLens 1® (Microsoft Corporation, Redmond WA, USA) was used. The device is a completely self-contained wearable computer in the format of an OST-HMD. It has 2–3 h of active battery time, allowing cordless operations. It is documented that the Microsoft HoloLens 1 features a fixed focal length of 2.0 m and a field of view (FOV) of 35° [21, 22]. In order to ensure the best mixed reality experience, the HoloLens RGB “live capture” camera underwent a calibration process where intrinsic parameters were optimized. The intrinsic parameters represent a projective transformation from the 3D camera’s coordinates into the 2D image coordinates. The extrinsic parameters (unadjusted) represent a rigid transformation from 3D world coordinate system to the 3D camera’s coordinate system.

Camera calibration was performed in OpenCV, and these parameters are generally used in several computer vision applications [23].

#### Software: “CTrue” software application

An original software application (app) was developed compatible with the Microsoft HoloLens 1^®^, and named “CTrue” version 1.0 (reads “see-through”). The software Unity (version:2018.3.8f1, Unity technologies, CA USA) and its Unity3D package was used to develop this HoloLens app. The purpose of the CTrue app is to localize structures hidden beneath the surface of the phantom. In this way, the CTrue app is capable of both uploading the 3D model (carrying orientating coordinate values) and registering it with the real world. During the registration step, the CTrue app uses the HoloLens “live capture” camera to detect the ArUco markers and register the 3D model on the phantom, 8 mm beneath its outer surface”.

The following software statistics were also used: OpenCV version: 4.0.0, Scripting backend: il2cpp, Target Windows SDK: 10.0.17763.0, Processor Architecture: ×86, Scripting Runtime Version: .NET 4.x equivalent, API Compatibility level: .NET Standard 2.0. and Visual Studio 2017 version: 15.9.10. All user interface elements and functionalities integrated in the “CTrue” app were developed by software engineers under direct supervision of medical end-users (surgeons).

#### Integration of fiducial (reference) markers as a registration system

Automatic registration (overlapping between physical and virtual objects) was achieved through the use of printed “ArUco” markers [24]. ArUco markers comprised of external squared black borders and an internal identification code (Fig. 1). This registration system was integrated in the software architecture of the “CTrue” app in order to be used during the experimental workflow. The RGB camera of the HoloLens captures the markers in the working field, in this way automatically obtaining the position of the markers in the real world. The CTrue application was engineered to detect and use five different ArUco markers (dimensions 40 × 40 mm) placed on corresponding surface points of the MRNOS phantom, as shown in Fig. 2f.

#### 3D model (hologram) creation and data upload

The MRNOS phantom was scanned in a iCT 256 computer tomography (CT) machine (Philips, Amsterdam; Netherlands) using standard protocols (1 mm slice thickness or lower) at the Department of Radiology of Akershus University Hospital. CT images were then exported to digital imaging and communications in medicine (DICOM) format and imported into the software 3D slicer version 4.10.1 (https://www.slicer.org). All 18 metal target crosses were segmented simultaneously into one single 3D model using thresholding image segmentation technique under the segment editor module in 3D slicer and later exported into stereolithography (STL) format. The 3D model was then converted into polygon (PLY) format using MeshLab version 1.3.3.1 [25] in order to be compatible with the CTrue app. With the same acquired CT dataset, ArUco markers then had to be identified on the DICOM images in order to acquire their digital coordinate values by using the *create*-*and*-*place* fiducial marker option in 3D slicer program. This allowed to record exact coordinate values on each plane in relationship with the CT volume data and export these values as a single file in JavaScript object notation (JSON) format. Both PLY and JSON files were then uploaded to a secure server which was linked to the CTrue app. The 3D model and fiducial marker coordinate values could be accessed instantaneously within the CTrue app while running on the HoloLens 1 device.

#### The variable

**Fig. 3:**
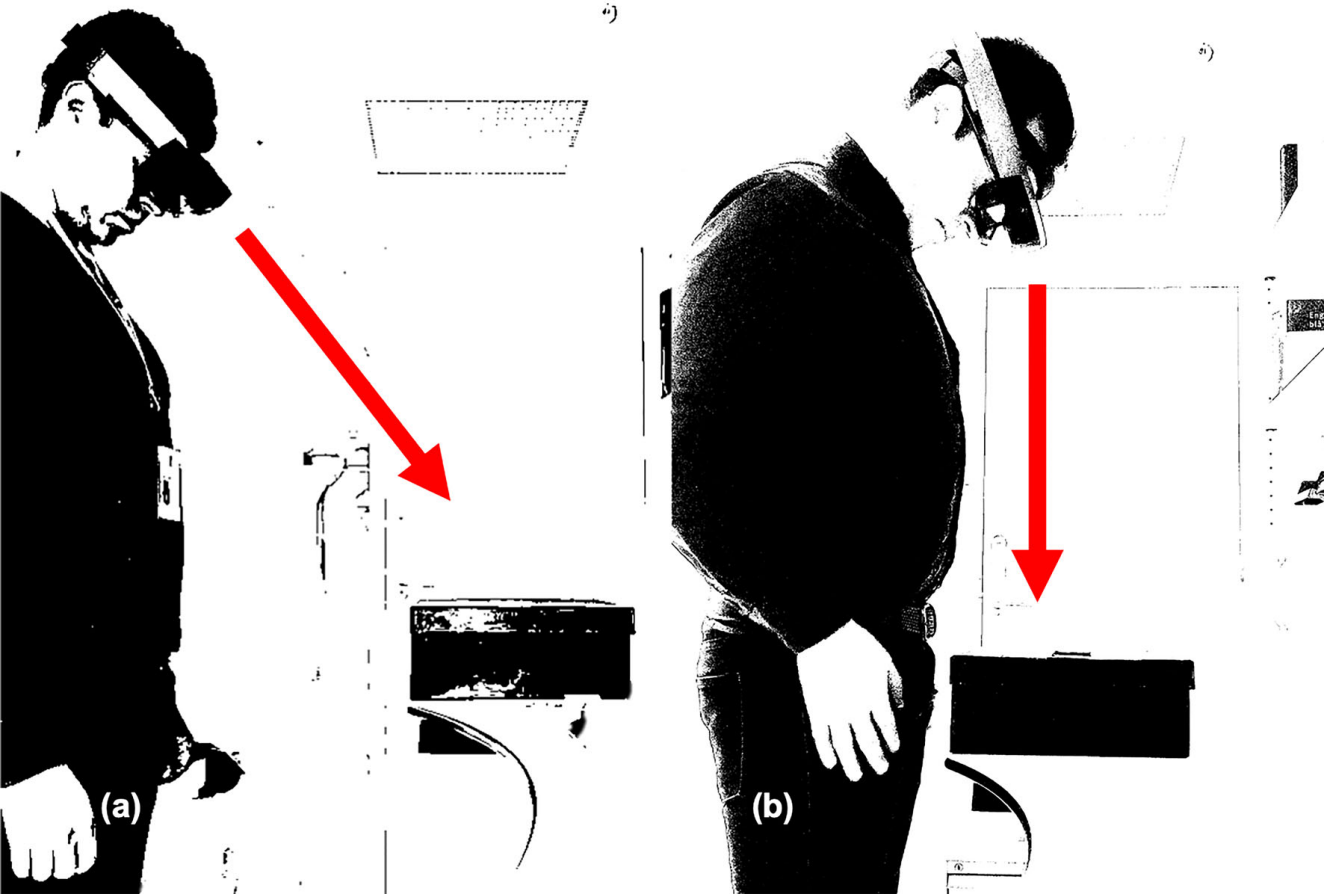
The surgeon’s sightline. Surgeon wearing the HoloLens and facing the MRNOS. **a** Represents the non-perpendicular free sightline/F/. **b** Represents the strictly perpendicular sightline/P/. Black and white effect to emphasize *the subject* and *the phantom*

The surgeon’s sightline (line of sight) is an imaginary and unobstructed straight line connecting the surgeon’s eye to the target [26]. For the purposes of this experiment, only two possibilities are provided for this variable: a strictly *perpendicular sightline* (P) upon the tested axis, and a *free sightline* (F), which required a constant but non-perpendicular sightline at the convenience of the surgeon (Fig. 3).

### Intraoperative workflow simulation

Six surgeons at Akershus University Hospital were invited on a voluntary basis to perform a predetermined *two*-*step* task divided in *four* different scenarios. The hospital’s internal review board (IRB) granted permission to conduct this experiment. After signed informed consent was secured, all subjects underwent an individual and standardized 15 min instruction session held by the first author (JAL) on how to use the HoloLens and to perform the experiment. None of the participants had previous experience with the HoloLens. Before the start of each test, a biometric calibration, the IPD (interpupillary distance), was performed by each subject by using the built-in “Calibration” app [27] on the HoloLens menu. Wearing glasses or contact lenses did not represent a physical obstacle while wearing the HoloLens.

Each subject performed (HoloLens worn and CTrue app launched) the following two main steps:

*Step 1 (Registration step)* to automatically place (register) the virtual model (hologram) so that it is superimposed with its real-world counterpart using the ArUco fiducial markers.

The subject using the HoloLens and the CTrue app had to look at the ArUco markers, placed on the *Y*-axis (anterior–posterior) of the phantom, during the registrations step. Immediately after this, a *registration sphere* appeared floating on each ArUco marker. This was an automatic quality control step where each sphere had to be located in the center point of the each ArUco marker in order for the app to register the 3D model inside the phantom, before being able to continue to the next *targeting step.*

*Step 2 (Targeting step)* Visualize the virtual model (registered inside the MRNOS phantom, Fig. 2f), choose the entrance point, angle and trajectory, at the outer wall of the phantom, for correctly placing the tip of the needle in the middle of the “projected virtual cross” inside the MRNOS phantom, thus perforating both the foam plate and the paper containing the physical target cross, simulating the perforation of mesenteric fatty tissue. In this step, subjects were neither able to change the entrance point nor the angle and trajectory of the needle once the entrance point was chosen, as the copper wire targets were 8 mm deep from the entrance point. This step was also performed under continuous verbal supervision.

Furthermore, four different scenarios affecting the registration and targeting were developed by controlling the *surgeon’s sightline,* while performing either step 1 or step 2.

The order of scenarios to be tested was predetermined as follows:

*Scenario FF* Free sightline during step 1 and step 2.

*Scenario PF* Perpendicular sightline during step 1 and Free sightline during step 2.

*Scenario FP* Free sightline during step 1 and Perpendicular sightline during step 2.

*Scenario PP* Perpendicular sightline during step 1 and step 2.

#### Registering and targeting with perpendicular sightline (P)

During registration, the researcher placed the test subject in a standing position, where the subject’s feet were not further than 15 cm away from the phantom (marked line on the floor). In addition, the distance between the HoloLens and the ArUco markers was held constant at around 70 cm during the registration step and controlled by using a height-adjustable table under the supervision of the first author (JAL). During targeting, the eye-target distance, consisting of the surgeon’s arm’s length (40–50 cm), was maintained among participants. Throughout both steps, a *perpendicular sightline* was strictly enforced and controlled for each of the three axes by continuous verbal instruction. The subject was also instructed to minimize his/her head movements.

#### Registering and targeting with free sightline (F)

During *free sightline* conditions, while the angle of the subject’s head was not controlled neither during registering nor targeting, the subject´s feet could not trespass a distance of 30 cm (marked line on the floor) in relationship with the outer border of the phantom during the registration step. Also, the distance between the HoloLens and the ArUco markers was held constant at around 70 cm during registration and controlled with the height-adjustable table. The eye-target distance was also maintained to arm's length (40–50 cm) during the targeting step.

This two-step experiment was repeated (attempt 1 and attempt 2) in each scenario, resulting in 8 datasets per participant and scenario, and data was collected first before moving on to the next scenario.

### Accuracy assessment

Each participant was given a total of 18 needles during each scenario. Six needles had to perforate into the phantom and correctly meet the middle of the copper wire cross targets (defined as the *bullseye*) on each axis (*XYZ*) using the hologram as a navigational cue.

The efficacy and performance of the “CTrue” app navigation system were assessed in terms of target error distance (TED). TED was defined as the distance from one side surface of the perforated needle to the same side midpoint of the copper wire cross, TED was measured in millimeters (mm) with a digital caliper on every target by the same person (JLA) (Fig. 2e). Any TED value between 0 and 0.5 mm was considered to have shot the *bullseye* throughout the data.

All 18 datapoints in each scenario had to be recorded before performing a second attempt and moving to the next scenario. The Microsoft HoloLens was reset after every data collection. This required closing the “CTrue” app from the operating system, turning off the HoloLens, removing the inserted needles from the phantom and repositioning the study subject accordantly before starting the two-steps task on each tested scenario.

### Statistical analysis

Statistical analysis was performed with SPSS statistics version 25 (IBM Corp. 2017) using descriptive statistics. ANOVA and the post hoc Tukeys HSD multiple comparison test were used to compare multiple scenarios and find any significance between mean TED values. Data analysis was divided by both axes (*X*, *Y* and *Z*) and scenarios (FF, PF, FP and PP) in order to understand the impact of both working axes and surgeon’s sightlines on registration and target accuracy. Paired sample *t* test was performed for analysis between repeated attempts. Violin plots were created using BioVinci Software (Bioturing, San Diego, CA, USA).

## Results

### Study subjects

Six surgeons (1 female, age range 29–62, median age 38) working at Akershus university hospital were enrolled in this study. One participant used correction glasses and three used correction contact lenses during the experiment. There were two cases of self-reported reduced stereopsis which could have affected depth perception during the experiment.

### Collected data

A total of 864 datapoints were collected from 6 subjects in 4 different scenarios, including one repeated attempt. Descriptive statistics from target error distances obtained in all four scenarios were grouped according to axis and reported in Table 1.

**Table 1:** Descriptive statistics

	N	Mean	SD	95% confidence interval for mean		Minimum	Maximum
				Lower bound	Upper bound		
*X*-axis							
Scenario FF	72	10.0275	3.18785	9.2784	10.7766	0.70	14.98
Scenario PF	72	7.2106	3.12192	6.4769	7.9442	1.55	13.67
Scenario FP	72	7.0639	3.44868	6.2535	7.8743	1.12	14.84
**Scenario PP**	**72**	**2.9757**	**1.33396**	**2.6622**	**3.2892**	**0.50**	**6.52**
*Y*-axis							
Scenario FF	72	6.3556	3.35753	5.5666	7.1445	0.50	12.84
Scenario PF	72	3.5036	2.05205	3.0214	3.9858	0.50	9.30
Scenario FP	72	2.8536	1.62539	2.4717	3.2356	0.50	6.22
**Scenario PP**	**72**	**2.2790**	**1.44992**	**1.9383**	**2.6197**	**0.50**	**6.61**
*Z*-axis							
Scenario FF	72	16.1132	8.91073	14.0193	18.2071	0.64	32.17
Scenario PF	72	13.0189	7.73185	11.2020	14.8358	0.65	30.70
Scenario FP	72	7.2893	4.06745	6.3335	8.2451	0.50	14.63
**Scenario PP**	**72**	**2.7844**	**1.91323**	**2.3349**	**3.2340**	**0.50**	**7.37**

Dataset divided in tested axes and sightline scenarios

Mean values represent all attempts performed. Highlighted values present the smallest target error distance (TED) observed. All values are measured in mm

**Fig. 4:**
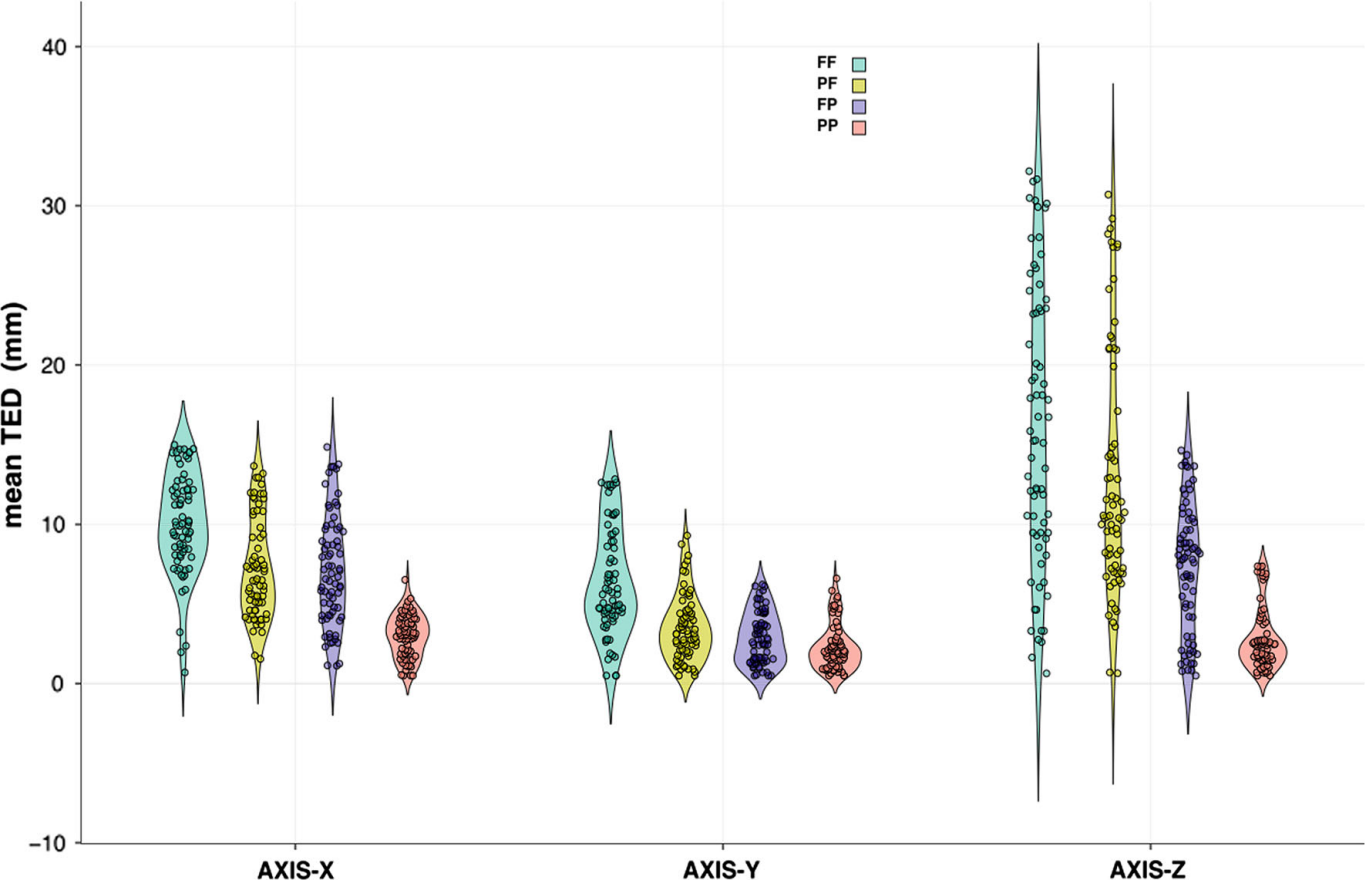
Violin plot showing the probability density of all datapoints. Target error distance (TED) in millimeters (mm). Data grouped in both scenarios (color-coded) and axes (*XYZ*). Dots represent datapoints

Scenario PP showed the smallest TED in *X*–*Y*–*Z* planes with mean = 2.98 mm ± SD 1.33; 2.28 mm ± SD 1.45; 2.78 mm ±SD 1.91, respectively. Scenario FF showed the largest TED in *X*–*Y*–*Z* planes with mean = 10.03 mm ± SD 3.19; 6.36 mm ± SD 3.36; 16.11 mm ± SD 8.91, respectively. Overview is shown in Fig. 4.

### Multiple scenario comparison

Prior to conducting the ANOVA test, the assumption of normality was evaluated and determined to be satisfied as the distributions on all four different scenarios were associated with skew and kurtosis less than 2.0 and 9.0, respectively (Table 1).

Multiple comparisons test results are shown in Table 2.

**Table 2:** Post hoc Tukey’s HSD multiple comparison between mean target error distance (TED) values

Multiple comparisons						
Tukey HSD	Dependent variable		Mean difference (*I*–*J*)	Sig.	95% confidence interval	
					Lower bound	Upper bound
*X*-axis	Scenario FF	Scenario PF	2.8169	0.000*	1.5689	4.0649
		Scenario FP	2.9636	0.000*	1.7156	4.2116
	Scenario PF	Scenario PP	7.0518	0.000*	5.8038	8.2998
		Scenario FP	0.1466	0.990	− 1.1013	1.3947
	Scenario FP	Scenario PP	4.2348	0.000*	2.9869	5.4829
		Scenario PP	4.0881	0.000*	2.8402	5.3362
*Y*-axis	Scenario FF	Scenario PF	2.8519	0.000*	1.8834	3.8205
		Scenario FP	3.5019	0.000*	2.5334	4.4705
	Scenario PF	Scenario PP	4.0765	0.000*	3.1079	5.0451
		Scenario FP	0.6500	0.308	− 0.3186	1.6186
	Scenario FP	Scenario PP	1.2245	0.007*	0.2560	2.1932
		Scenario PP	0.5745	0.419	− 0.3940	1.5432
*Z*-axis	Scenario FF	Scenario PF	3.0943	0.018*	0.3754	5.8132
		Scenario FP	8.8238	0.000*	6.1050	11.5428
	Scenario PF	Scenario PP	13.3287	0.000*	10.6099	16.0476
		Scenario FP	5.7295	0.000*	3.0107	8.4485
	Scenario FP	Scenario PP	10.2344	0.000*	7.5156	12.9533
		Scenario PP	4.5048	0.000*	1.7860	7.2237

Data is grouped in tested axis and tested scenarios. Mean TED values in mm. *Represent statistically significance values in mean difference (*p *< 0.05). Dataset includes both attempts

*X*-*-axis* The mean of one comparison set was not significantly different (scenarios PF and FP, *p* = 0.990) while all other possible mean comparisons were significantly different (*p* < 0.05).

*Y*-*-axis* The mean of two comparison sets were not significantly different (Scenarios PF-FP, *p* = 0.308 and scenarios FP-PP, *p* = 0.419) while all other possible mean comparisons were significantly different (*p* < 0.05).

*Z*-*-axis* All possible mean comparisons among four scenarios were significantly different (*p* < 0.05). Multiple scenario comparison dataset contains all repeated attempts.

### Repeated attempts analysis

**Fig. 5:**
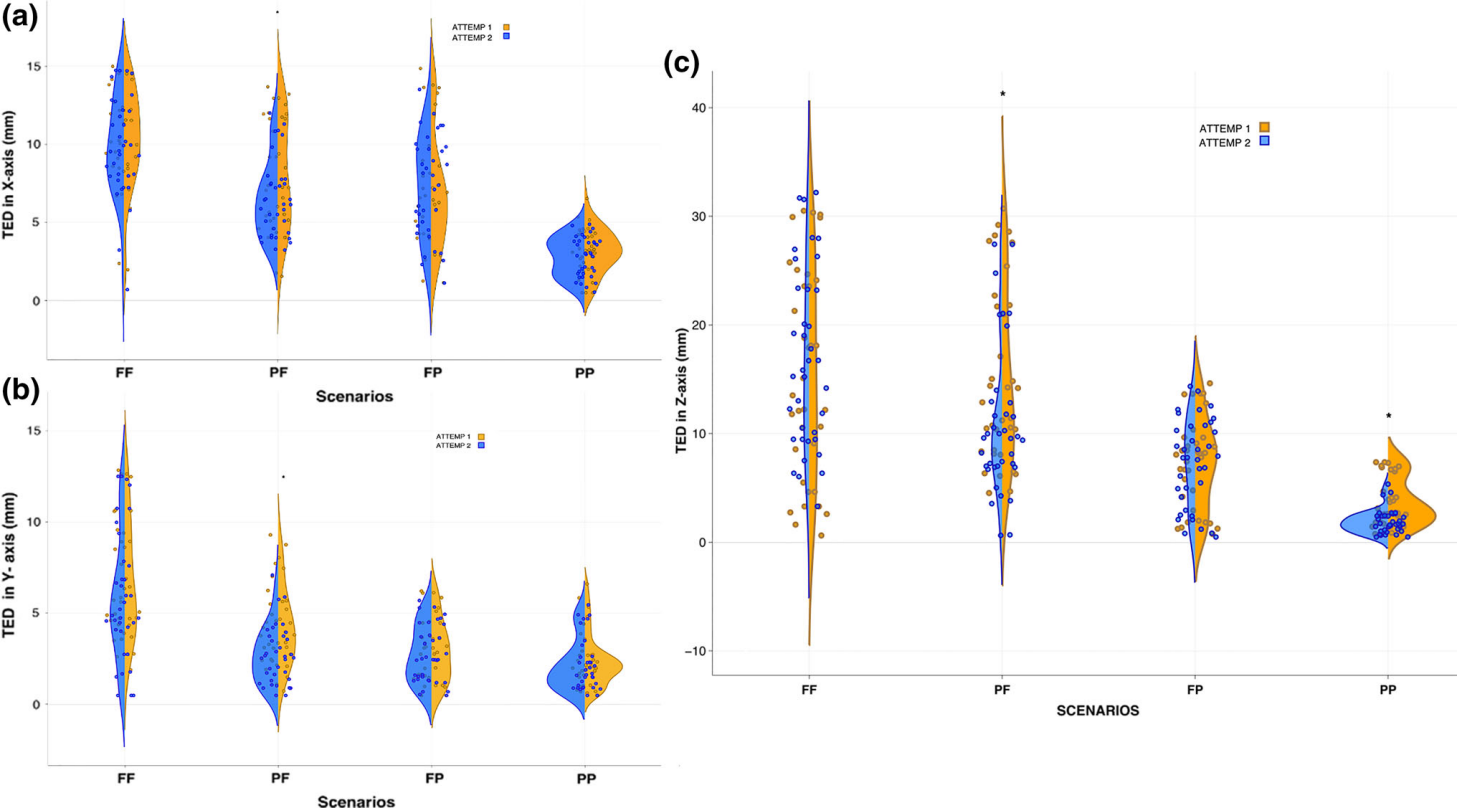
Violin plot of data divided between attempt 1 and 2. Target error distance (TED) in millimeters (mm). **a** Values in *X*-axis grouped in 4 different scenarios. **b** Values in *Y*-axis grouped in 4 different scenarios. **c** Values in *Z*-axis grouped in 4 different scenarios. Asterisk represents statistically significant differences between attempt 1 and 2

Attempt 2 showed a significantly lower mean TED value in relationship with attempt 1 (*p* = 0.028, *df* = 11, *t* = 2.531) in overall data. When data were grouped by specific scenario, PF showed a significant reduction of mean TED values for all axes during the second attempt. These were as followed: *X*-axis improved from 7.94 mm to 6.49 mm (*p* < 0.05), *Y*-axis from 4.29 mm to 2.71 mm (*p* < 0.05) and *Z*-axis from 15.31 to 10.73 mm (*p* < 0.05). In addition, attempt 2 showed a significant reduction of mean TED in scenario PP only on the *Z*-axis. Otherwise, there were no statistical differences between mean TED values in all axes when scenarios FF, FP and PP were repeated. *p* values showed in Table 3. Violin plot of data is shown in Fig. 5.

**Table 3:** Repeated attempts analysis

Mean TED values	Attempt 1	Attempt 2	*p* value (one-sided paired *t* test)	
Scenario FF				
*X*-axis	10.31	9.75	0.21	
*Y*-axis	6.82	5.89	0.05	
*Z*-axis Total completion time (m)	15.80 03:05	15.43 03:01	0.27	
Scenario PF				
*X*-axis	7.94	6.49	0.007	*
*Y*-axis	4.29	2.71	0.000	*
*Z*-axis Total completion time (m)	15.31 02:42	10.73 02:20	0.000	*
Scenario FP				
*X*-axis	6.87	7.26	0.32	
*Y*-axis	2.97	2.74	0.29	
*Z*-axis Total completion time (m)	7.25 02:25	7.33 02:05	0.45	
Scenario PP				
*X*-axis	3.14	2.81	0.14	
*Y*-axis	2.41	2.15	0.14	
*Z*-axis Total completion time (m)	3.66 01:46	1.91 01:44	0000	*
Mean TED values (mm) (total sum)	7.23	6.27	0.028	*
Mean completion time (m) (total sum)	02:29	02:17	0.052	

Data grouped in sightline scenarios and tested axis

target error distance (TED) values are in millimeters (mm). Completion time are in minutes (m). *Represent statistically significant values (*p *< 0.05)

### Time measurements

Median *registration time* among all participants was 0.9 min, range 00:19–02:01 min (m) (± SD 0.022). Median *target*ing *time* among all participants was 01:29 m, range 1:20–1:40 m (± SD 0.0036). Total time to perform both experimental steps reached a median of 02:23 m, range 01:44–03:26 m (± SD 0.021). No statistical difference was found between total completion time between attempt 1 and 2 (*p* = 0.052), regardless of scenario.

## Discussion

**Fig. 6:**
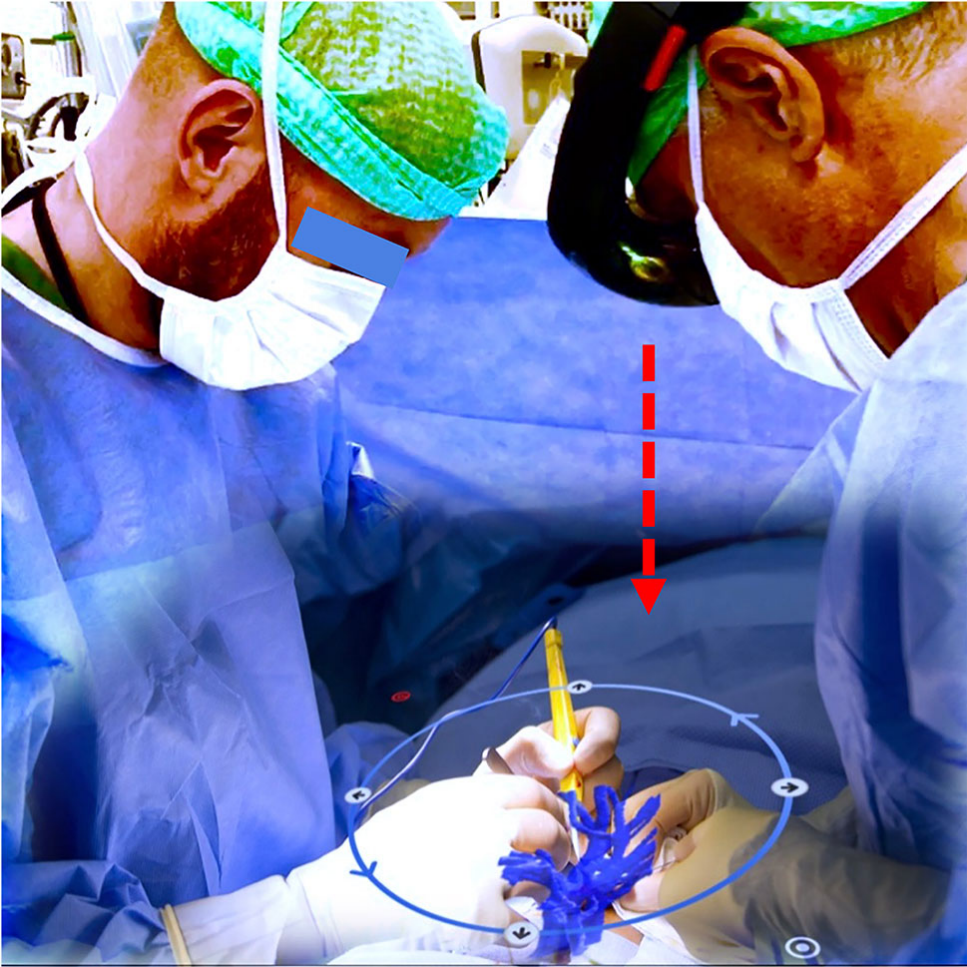
Graphical simulation of clinical application. Surgeon wears the Microsoft HoloLens 1 and uses the CTrue app, which projects a CT-derived 3D vascular model into the mesentery for open surgery navigation. Red arrow represents the surgeon’s perpendicular sightline

The most important finding of this study is that a strictly perpendicular surgeon´s sightline (as previously defined) has a highly significant impact on both 3D model registration and targeting accuracy while using the inside-out marker-based MRNOS. When the surgeon used a strictly perpendicular sightline in both registration and targeting (PP), the smallest TED was consistently observed on all axes, as presented in Fig. 4. In addition, this 2 mm error range was achieved without adding any commercial outside-in optical tracking systems, in comparison with other studies achieving similar results [1, 17, 28]. While different studies testing the accuracy of ArUco marker-based registration can be found in the literature [24, 29], the units of accuracy are not usually given in mm, but rather in pixels and computing time because the camera used in the MR device has a major role in its registration accuracy. There are, however, some ArUco-based MR registration studies using the HoloLens that give accuracy in ranges from 0.47 mm to 2.3 cm [30, 31]. Our results fall in this range. On the other hand, our results from the FF scenario on the *Y*-axis (TED values close to the 6 mm range) corroborate with results obtained by Condino et al. [8]. These results emphasize the importance for sightline awareness and correction when using the HoloLens glasses at surgery (Fig. 6).

Overall, the literature presents error ranges from 0 to 10 mm by using diverse navigation and accuracy measurement methods on different surgical specialties with the Microsoft HoloLens [8, 28, 29, 32–34]. This collective experimental heterogeneity makes it cumbersome to pinpoint the crucial elements that create such data variability. Nevertheless, from a technical perspective, it is important for the clinician to realize that several error sources can exist simultaneously (user-induced and computational). Among user-induced error sources, different degrees of visual acuity and depth perception (stereopsis) can influence target accuracy [35]. While only interpupillary distance (IPD) calibration was performed here, other biometric parameters cannot be underestimated and should be taken into account.

Our results indicate an obvious advantage in the *Y*-axis showing the smallest TED values in all sightline scenarios, most noticeably in scenario PP. A plausible explanation is that the registration step was exclusively done using the *Y*-axis, as this is the axis where the ArUco markers were placed on the phantom. Incidentally, the *Y*-axis, seen with a perpendicular sightline, also represents the natural working position of the open surgeon during the targeting step. While there is a possibility that the accuracy in *Y*-axis is better due to a positional preference from the surgeon, other factors as camera quality and calibration in any MR device play an important role [7]. What we also see in Table 2 is that the *Z*-axis contains larger mean differences for all tested scenarios, when comparing to *X*- and *Y*-axis. Regardless of scenario comparison, this table shows that accuracy is inferior mainly when performing the experiment in the *Z*-axis. Similar results concerning the *Z*-axis are also observed in previous studies [29, 36, 37]. To our knowledge, there are no previous works in the literature emphasizing the importance of visual axes on TED in an inside-out, marker-based navigation system and demonstrating consistent variations in registration accuracy. What can be found in the literature are data on outside-in tracking systems and/or marker-less surface geometry tracking systems that corroborate with our results, although not showing accuracy in same axes [1, 17, 38]. Clinically, we can suggest that there is a greater value in using the HoloLens only in small time intervals. Mainly, we believe that the hologram can potentially obstruct the view of the tissue to be operated when used for long periods of time [39]. In addition, our results let us imply that it is important to understand how surgeons tend to work in a “nearly” perpendicular position when performing a laparotomy. This means that the surgeon will only have to accommodate to a “strictly” perpendicular sightline, while using the HoloLens, *only* when anatomical orientation is essential throughout the procedure. This “*on and off”* principle also fits with the new design of the Microsoft HoloLens 2 having a “flip-up” visor which encourages only temporary use of the HoloLens [40].

From the perspective of colon cancer surgery, future navigation technologies aim to help democratize patient-tailored surgery. In this way, MR devices have the potential to facilitate the introduction of complex surgical procedures, which the surgical community is typically reluctant to implement, as standard treatment options [13]. The ultimate goals are to both avoid devastating vascular injuries and to improve overall outcomes in cancer patients [12, 19].

Analysis of our results indicates that such systems can provide anatomical awareness to operating surgeons performing central mesenteric lymphadenectomy as it is precise enough to help identify *the initial incision* in the mesentery, as well as to localize the pathology, and also to continue with any remaining vascular dissection. This becomes more obvious when it is understood that the mesenteric vessels are embedded in the mesenteric fat and not readily visible at surgery [41]. One should not forget that such systems can similarly provide confidence to the operating surgeon by assisting in the identification of a bleeding vessel, with good enough precision, when hemostasis is required. For these reasons, the MRNOS was constructed as a controlled environment to prove feasibility by simulating mean values of mesenteric fat thickness, allowing the placement of virtual targets at a depth of 8 mm from its outer surface. In addition, we must highlight that the root of the mesentery, and its vasculature, is fused to the retroperitoneum, thus reducing the need for deformable 3D models [42].

At first glance, the number of participants included in this study can be considered a study limitation. However, each participant created a total of 36 datapoints in 2 repetitive rounds thus creating a total of 864 analyzed datapoints throughout the study. Moreover, participating surgeons reported different holographic perceptions, either by not realizing that the 3D model was deep *inside* the phantom, and also noticing different degrees of visual occlusion, when finding the correct targeting point, due to the level of brightness from the 3D model. The visual perception of the 3D model was improved by individually adjusting the 3D model’s transparency with the *brightness bottom* on the HoloLens (less brightness from the 3D model produced more transparency, thus better visibility on the bullseye).

It is also important to add that the perpendicular registration and targeting (PP) scenario had to be placed at the end of scenarios sequence in order to prevent bias, where the subjects could have learned that the perpendicular sightline gave better visual aid earlier in the experiment. For this reason, the scenario order could not be randomized.

Moreover, Table 3 shows significant improvement of total TED values in attempt 2 when compared to attempt 1, regardless of scenario. We also find that each experimental round was not time consuming, not taking more than 4 min, where similar timing has also been presented in other studies [32, 43]. Table 3 also presents that there was no statistical difference in completion time between the two attempts.

Following the IDEAL framework of surgical innovations [44], this work encourages us to move forward from the exploratory stage 2 to the assessment stage 3 with the aim to measure comparative effectiveness with current clinical workflows. Finally, with rapid technological advances, surgeons must play a more active role in the assessment and implementation of new navigation systems and ensure that such innovations will meet the surgeon’s (and the patient’s) needs. This also entails the role to assess various 3D anatomical format, physical or virtual, which are the main ingredients of any surgical navigation system [45].

## Conclusion

In conclusion, we show that the value of the surgeon’s sightline on registration and targeting accuracy, while using an inside-out marker-based mixed reality navigation system for open surgery, is significant, while precision, on both tasks, is also axis-dependent. Coincidentally, best results (2.28 mm accuracy on the *Y*-axis) are achieved using the natural line of sight of the open surgeon, implying that it is precise enough to test in a clinical setting.
